# Metabolomic profiling and antibacterial efficacy of probiotic-derived cell-free supernatant encapsulated in nanostructured lipid carriers against canine multidrug-resistant bacteria

**DOI:** 10.3389/fvets.2024.1525897

**Published:** 2025-01-03

**Authors:** Nay Zin Myo, Ratchnida Kamwa, Thitirat Jamnong, Busaba Swasdipisal, Papavarin Somrak, Phanchompoo Rattanamalakorn, Vipada Neatsawang, Prasert Apiwatsiri, Teerapong Yata, David J. Hampson, Nuvee Prapasarakul

**Affiliations:** ^1^Department of Veterinary Microbiology, Faculty of Veterinary Science, Chulalongkorn University, Bangkok, Thailand; ^2^The International Graduate Course of Veterinary Science and Technology, Faculty of Veterinary Science, Chulalongkorn University, Bangkok, Thailand; ^3^Department of Veterinary Biochemistry, Faculty of Veterinary Science, Chulalongkorn University, Bangkok, Thailand; ^4^School of Veterinary Medicine, Murdoch University, Perth, WA, Australia; ^5^Center of Excellence in Diagnostic and Monitoring of Animal Pathogens, Faculty of Veterinary Science, Chulalongkorn University, Bangkok, Thailand

**Keywords:** antibacterial activity, probiotic cell-free supernatant, nanostructured lipid carriers, metabolomic analysis, *Pseudomonas aeruginosa*, *Staphylococcus pseudintermedius*

## Abstract

**Aim:**

This study aimed to investigate the antibacterial efficacy of probiotic-derived cell-free supernatants (CFS) encapsulated within nanostructured lipid carriers (NLCs) against multidrug-resistant *Pseudomonas aeruginosa* and *Staphylococcus pseudintermedius*. Additionally, it aimed to identify specific bioactive compounds that contribute to the reported antibacterial properties by characterizing the metabolite substances present in the CFS using a metabolomic analysis technique.

**Methods:**

Eight strains of lactic acid bacteria including *Lactiplantibacillus plantarum* (L22F and L25F), *Pediococcus acidilactici* (P72N, BF9, BF 14, BYF 20 and BYF 26) and *Ligilactobacillus salivarius* (BF 12) were selected as probiotic candidates. The inhibitory activity of their cell free supernatant (CFS) was tested against clinical strains of *P. aeruginosa* and *S. pseudintermedius* isolated from skin wounds of dogs and cats. An untargeted metabolomic approach based on liquid chromatography-mass spectrometry (LC-MS) identified potential antibacterial metabolites in the CFS. Cell-Free Supernatants-Nanostructured Lipid Carriers (CFS-NLCs) were developed, and their antibacterial activity and minimum bactericidal concentration (MBC) were analysed.

**Results:**

Despite the strong multidrug-resistant nature of the pathogens, CFS displayed a moderate antibacterial activity against most tested strains. The acidic nature of the CFS, combined with bioactive antibacterial metabolites like Kanzonol V and 1-Hexanol, likely contributed to its inhibitory effects against pathogenic bacteria; notably, Kanzonol V was abundant in the CFS of L22F, BF12 and BYF26 (L22F_CFS, BF12_CFS and BYF26_CFS), while 1-Hexanol was particularly enriched in CFS of P72N (P72N_CFS), with both compounds effectively targeting bacterial cell membranes to disrupt cell integrity, leading to bacterial cell death. Other beneficial compounds such as Pyroglutamylleucine, Trigoneoside VIII and 18-Nor-4(19),8,11,13-abietatetraene which are likely to have anti-inflammatory, antimicrobial and antioxidant activities, were also detected in the CFS. The CFS-NLCs maintained their antibacterial activity and 30–60% dilutions of product completely inhibited the growth of pathogen strains even after three-months storage at room temperature.

**Conclusion:**

These findings suggest that CFS-NLCs could be a promising biotic therapy for treating hospital infections such as canine dermatitis and otitis caused by multidrug-resistant *P. aeruginosa* and *S. pseudintermedius*.

## Introduction

The global increase in antibiotic resistance among bacteria presents a significant challenge, with multidrug-resistant (MDR) pathogens like *Pseudomonas aeruginosa* and *Staphylococcus pseudintermedius* becoming increasingly difficult to treat. *P. aeruginosa* is an opportunistic pathogen commonly associated with hospital-acquired infections, particularly in immunocompromised patients, while *S. pseudintermedius* is primarily a veterinary pathogen but can also cause zoonotic infections in humans (1, 2). These pathogens are notorious for their role in causing dermatitis and otitis, conditions that can severely affect the health and quality of life in dogs and cats (3, 4). Both pathogens exhibit extensive resistance to conventional antibiotics, complicating treatment and increasing morbidity and mortality rates. The urgent need for alternative antimicrobial strategies has driven research into novel therapeutic approaches, including the use of natural bioactive compounds (5).

Probiotics, live microorganisms that confer health benefits to the host, are known not only for their role in gut health but also for their production of bioactive metabolites. The specific metabolic profiles of probiotic-derived cell free supernatant (CFS) encompass a variety of organic acids, bacteriocins, and other bioactive compounds that collectively contribute to their antimicrobial activity against pathogenic bacteria (6). Metabolomic analysis using liquid chromatography–tandem mass spectrometry (LC–MS/MS) is a robust method for determining the composition of metabolites in CFS derived from probiotics. These techniques provide insight into the metabolic profiles of probiotic strains and an understanding of how different metabolites contribute to the observed antimicrobial effects. Understanding these metabolic contributions is essential for developing effective biotic therapies for managing infections in veterinary medicine and beyond (7, 8). Some studies have highlighted the potential use of probiotic-derived CFS as a biotic therapy to inhibit the growth of pathogenic bacteria (9, 10). However, the instability and rapid degradation of these bioactive compounds inside the CFS *in-vivo* limit their clinical application, necessitating the development of novel delivery systems to enhance their efficacy.

Nanostructured lipid carriers (NLCs) have emerged as a promising delivery platform for bioactive compounds due to their ability to protect sensitive molecules, improve their stability, and enhance controlled release. NLCs, composed of a mixture of solid and liquid lipids, offer superior biocompatibility and bioavailability (11), making them an ideal carrier for probiotic-derived metabolites. The current study aimed to investigate the antibacterial activity of probiotic-derived CFS encapsulated in nanostructured lipid carriers (NLCs) against multidrug-resistant *P. aeruginosa* and *S. pseudintermedius.* Additionally, by employing a metabolomic analysis technique to characterize the metabolite substances in the CFS, it sought to pinpoint bioactive compounds with antimicrobial activity.

## Materials and methods

### Lactic acid bacteria sources and culture conditions

Eight lactic acid bacteria (LAB) strains were selected from the culture collection held at the Faculty of Veterinary Science, Chulalongkorn University for use in the study. These included *Lactiplantibacillus plantarum* 22F (L22F)*, L. plantarum* 25F (L25F) and *Pediococcus acidilactici* 72 N (P72N) which were isolated from the feces of pigs and *Ligilactobacillus salivarius* BF12 (BF12), *P. acidilactici* BF9 (BF9), *P. acidilactici* BF14 (BF14), *P. acidilactici* BYF20 (BYF20), and *P. acidilactici* BYF26 (BYF26) which were isolated from the feces of chickens. All eight strains have been suggested as potential probiotic candidates in previous studies (12, 13). The bacteria were cultivated on de Man Rogosa Sharpe (MRS) agar (Becton, Dickson and Company, Maryland, United States) and incubated at 37°C for 48 h. A matrix-assisted laser desorption/ionization time-of-flight (MALDI-TOF) Biotyper (Bruker Daltonics, MA, United States) then was used to confirm the identity of each strain. All sample collections were approved according to the guidelines for experimental animals established by the Chulalongkorn University Institute Animal Care and Use Committee (agreement no. IBC20310148).

### Cell free supernatant preparation

Five to ten colonies of each LAB strain from freshly cultured plates were subcultured into 10 mL MRS broth and incubated at 37°C for 18 h. After incubation, the optical densities of the cell cultures were measured by spectrophotometer at a 600 wavelength and adjusted with sterile MRS broth to a density that corresponded to 10^8^ colony-forming units per milliliter (CFU/mL). The bacterial inoculum was added to the desired amount of MRS broth at a ratio of 1:100 and incubated at 37°C for 24 h. After incubation, the cell cultures were centrifuged at 4,500 rpm, 4°C for 10 min; the cell pellets were discarded, and the CFSs were collected. The pH values of the CFSs were recorded before sterile filtration at room temperature with 0.22-μm surfactant-free cellulose acetate filters (Corning, New York, United States).

### Pathogenic bacterial strains and bacterial inoculum preparation

Thirteen strains of *P. aeruginosa* (clinical strains 93, 1383, 1826, 1846, and 2054) and *S. pseudintermedius* (clinical strains 130, 159, 531, 668, and 998) were selected from frozen stock as indicator pathogens to test the antibacterial activity of probiotic CFS. These pathogens came from skin wounds of dogs and cats, and the sources of samples and origin (host) are presented in Supplementary Table S1. They were considered as multi-drug-resistant bacteria according to antimicrobial susceptibility test results using the Automated Vitek^®^ 2 Compact system (bioMérieux, France). The antibiogram of *S. pseudintermedius* strains and *P. aeruginosa* strains are presented in Supplementary Tables S2, S3, respectively. *P. aeruginosa* ATCC 27853, *S. pseudintermedius* ED99 and *S. aureus* ATCC 25923 were used as reference strains. The bacteria were subcultured on blood agar plates and then incubated at 37°C for 24 h. Subsequently, 1–2 colonies were selected and transferred into a desired volume of 0.85% NaCl solution. The cell suspensions were vortexed, and their densities were checked against McFarland standards to correspond to 10^8^ CFU/mL. These adjusted cell suspensions were used as basic inoculum for further determination of antibacterial activity and minimum bactericidal concentration (MBC).

### Determination of the antibacterial activity of probiotic cell-free supernatant

Using 100% crude cell free supernatant extracted from probiotic strains, the antibacterial activity against 13 selected bacterial pathogen strains was evaluated by the agar well diffusion method (14). The surfaces of Mueller-Hinton agar (MHA: Becton, Dickson and Company, Maryland, United States) plates were swabbed with cultures of the pathogen strains adjusted to approximately 10^7^ CFU/mL. Hundred microliter volumes of crude CFS were used to fill 8-mm diameter wells made in MHA. MRS and acidified MRS (pH 3.8 ± 0.05) were used as negative and positive controls, respectively. The plates were incubated at 37°C for 24 h, after which the diameter of the inhibition zone around the well was measured in millimeters and interpreted as (−) no inhibition, (+) mild inhibition (< 12 mm), (+ +) moderate inhibition (12–17 mm), and (+ + +) strong inhibition (> 17 mm).

### Exploration of bioactive metabolites in the cell-free supernatant of probiotic strains

#### Sample preparation for metabolomic analysis

When LAB strains are grown in water-based and highly salted environments they may produce lipids and proteins which potentially can cause interference in metabolomic analysis. To remove these, Oasis^®^ HLB SPE cartridges (60 mg, 3 mL; Waters, Milford, MA, United States) were prepared by conditioning with 1 mL methanol, followed by equilibrating with 1 mL of water. Five milliliter of CFS from each LAB strain cultivated in MRS medium, and sterile media (Blank), was centrifuged at 5,000 rpm for 10 min at 10°C, following which 2 mL of the upper layer was carefully collected and subjected to HLB-SPE purification. Each cartridge was washed with 1 mL water and metabolites were eluted with 1 mL methanol. The methanolic extracts were stored at −80°C until analysis (15).

#### Ultra-high-performance liquid chromatography coupled with electrospray ionization quadrupole time-of-flight mass spectrometry analysis

The instrument platform for this analysis was an ultra-high performance liquid chromatography coupled with electrospray ionization quadrupole-time-of-flight-mass spectrometry (UHPLC-ESI-Q-TOF-MS) (Bruker’s Compact). Separation was performed using a UHPLC system (Elute UHPLC, Bruker, Darmstadt, Germany) with the Bruker intensity solo HPLC column (C18 2.1 × 100 mm, 2 μm). The column temperature was set at 55°C and the autosampler temperature was set at 10°*C. mobile* phase A was 100% HPLC grade water, and mobile phase B was 100% methanol, with both containing 0.1% formic acid (FA). The flow rate was set at 0.4 mL/min and the elution gradient was set as—99.9% A (0.0–2.0 min, 0.25 mL/min), 99.9–75% A (2.0–10.0 min, 0.4 mL/min), 20% A (10.0–12.0 min, 0.4 mL/min), 10% A (12.0–21.0 min, 0.4 mL/min), 0.1% A (21.0–23.0 min, 0.4 mL/min), 99.9% A (24.0–26.0 min, 0.4 mL/min). A sample injection volume of 4 μL was applied for positive ionization polarity modes. 70% methanol was used as blank.

Mass spectrometry analyses were performed using a Compact ESI-Q-TOF system (Bruker, Darmstadt, Germany), and mass spectral signals were collected in positive ion scanning modes. Sodium formate (HCOONa) containing 2 mM sodium hydroxide, 0.1% FA and 50% IPA was directly injected as an external calibrant with a flow rate 0.05 mL/min. The conditions in the positive ionization polarity mode include mass range 50–1,300 m/z, capillary voltage 4,500 V, dry temperature 200°C, nebulizer 0.5 bar, and dry gas flow 4 L/min. The different datasets were analyzed using multivariate data analysis. One-way analysis of variance (ANOVA) combined with principal components analysis (PCA) and partial least squares discriminant analysis (PLS-DA) was used to evaluate the differential metabolites among control and sample groups. The analytical software used was from the online platform MetaboAnalyst 5.0.^1^

### Development of cell-free supernatant-nanostructured lipid carriers

Based on their antibacterial activity, the CFS from four of the eight strains (L22F, P72N, BYF26, and BF12) were chosen for the development of nano-encapsulation prototypes. CFS-encapsulated nanostructured lipid carriers (CFS-NLCs) were prepared using a hot homogenization method followed by ultrasonication. The oil phase consisted of a CFS-in-oil emulsion, prepared by mixing 79% (w/w) cell-free supernatant (CFS) and 10% (w/w) medium-chain triglycerides (MCT). This mixture was heated to 40–45°C with stirring until a stable emulsion was formed. The resulting emulsion was then heated to 70–75°C, followed by the addition of 3% (w/w) sorbitan oleate (Span 80). The mixture was stirred at the same temperature until the solid lipid melted completely and was homogeneously mixed with the liquid lipid phase.

The aqueous phase was prepared by dissolving 2% (w/w) poloxamer 188, 3% (w/w) polysorbate 20, 2% (w/w) glycerin and phenoxyethanol in distilled water. This solution was heated to 70–75°C under constant stirring to ensure complete dissolution and to match the temperature of the oil phase. Subsequently, the hot oil phase (20% of the total formulation) was added dropwise to the hot water phase (80% of the total formulation) under vigorous stirring to form a coarse emulsion. The coarse emulsion was subjected to ultrasonication using a probe sonicator set at 60–80% amplitude. Ultrasonication was performed in pulse mode (10 s on, 5 s off) for 3–5 min to achieve particle size reduction and improve emulsion homogeneity. The finished product was a nanostructured lipid containing CFS that did not separate into layers when maintained at room temperature (16). The CFS-NLCs was then stored at 4°C until further use.

### Measurement the physical characteristics of CFS-NLCs

The particle size and zeta potential of colloidal nanoparticles are critical parameters for assessing the efficacy of encapsulation in various applications. The properties of the NLCs achieved these two criteria, indicating efficacy and stability. Both these attributes of the colloidal nanoparticles were measured at 25°C using dynamic light scattering (DLS) (Nanosizer, Malvern, United Kingdom) following particle synthesis. The sample was diluted until 20 μL, and the volume was adjusted by adding water to a net volume of 1 mL.

### Determination the antibacterial activity of CFS-NLCs

The antibacterial activity of the 100% crude CFS-NLCs against 13 selected bacterial pathogens was rechecked using the same agar well diffusion method as earlier described in the “Determination the antibacterial activity of probiotic cell free supernatant” section. The same 100 μL volume of CFS_NLCs was used and CFS-free-NLC served as a negative control.

### Determination the minimum bactericidal concentration of CFS-NLCs

The MBC was determined using a protocol adapted from Pelyuntha et al. (17) and Taheur et al. (18). CFS-NLCs were mixed with pathogen strains in 96-well plates. Initially, 100% CFS-NLCs were serially diluted in Mueller-Hinton broth (MHB) (Becton, Dickson and Company, Maryland, United States) to reach a final concentration ranging from 10 to 90% (v/v), and 150 μL of CFS-NLCs were added to each well and mixed with 50 μL of each pathogen strain. Each well had a total volume of 200 μL and the final concentration of pathogen strain was 10^5^ CFU/mL. The MBC was calculated using the trial with the lowest percentage of complete inhibition of bacterial growth on solid media.

### Data analysis

The agar well diffusion assay and MBC test were performed in triplicate. For metabolomic data analysis, one-way analysis of variance (ANOVA) was used to investigate the differences in metabolites in each control and sample groups. Values were considered significant at *p* < 0.01. The statistical analysis was conducted using SPSS version 27.

## Results

### Antibacterial activity of the cell free supernatant of probiotics

The CFSs of all the tested probiotic strains were acidic, with the values for L22F, L25F, P72N, BF12, BF9, BF14, BYF20 and BYF26 being 3.82 ± 0.10, 3.79 ± 0.30, 3.78 ± 0.15, 3.89 ± 0.13, 3.84 ± 0.12, 3.74 ± 0.14, 3.77 ± 0.13 and 3.76 ± 0.40, respectively. The antibacterial activities of the CFS against *P. aeruginosa* and *S. pseudintermedius* in the agar well diffusion assay is presented Table 1. The MRS medium showed no inhibitory activity, whereas acidified MRS exhibited mild inhibition against all tested pathogenic strains. The CFS extracted from all probiotic strains exhibited moderate inhibition against all *P. aeruginosa* strains. In contrast, CFS from all probiotic strains displayed mild to moderate inhibition against most *S. pseudintermedius* strains. An exception was L25F-CFS, which showed mild inhibition only against *S. pseudintermedius* strain 998. Based on these antibacterial results, four probiotic strains including *Lactiplantibacillus plantarum* (L22F), *Ligilactobacillus salivarius* (BF12) and *Pediococcus acidilactici* (P72N and BYF26) were selected as representative strains for further metabolomic analysis and development of nano-encapsulation prototypes.

**Table 1:** Antibacterial activity of the cell free supernatant (CFS) from probiotic strains against strains of *P. aeruginosa*, *S. pseudintermedius* and *S. aureus* ATCC25923.

LAB CFS	*P. aeruginosa*						*S. pseudintermedius*						*S. aureus*
	93	1846	1826	1,383	2054	ATCC27853	159	130	531	668	998	ED99	ATCC25923
MRS	−	−	−	−	−	−	−	−	−	−	−	−	−
Acidified MRS	+	+	+	+	+	+	+	+	+	+	+	+	+
L22F	++	++	++	++	++	++	+	+	−	+	++	+	++
L25F	++	++	++	++	++	++	−	−	−	−	+	−	+
P72N	++	++	++	++	++	++	++	+	++	+	++	+	++
BF12	++	++	++	++	++	++	+	++	+	+	++	++	++
BF9	++	++	++	++	++	++	+	+	++	+	++	+	+
BF14	++	++	++	++	++	++	+	+	++	+	++	+	++
BYF20	++	++	++	++	++	++	+	+	++	+	++	+	++
BYF26	++	++	++	++	++	++	+	+	++	+	++	+	++

The diameter of the inhibition zone was measured in millimeters and interpreted as (−) no inhibition, (+) mild inhibition (< 12 mm), (+ +) moderate inhibition (12–17 mm), (+ + +) strong inhibition (> 17 mm).

### Metabolomic analysis by UHPLC-ESI-QTOF-MS

#### Determination of whole metabolite profiles

**Figure 1:**
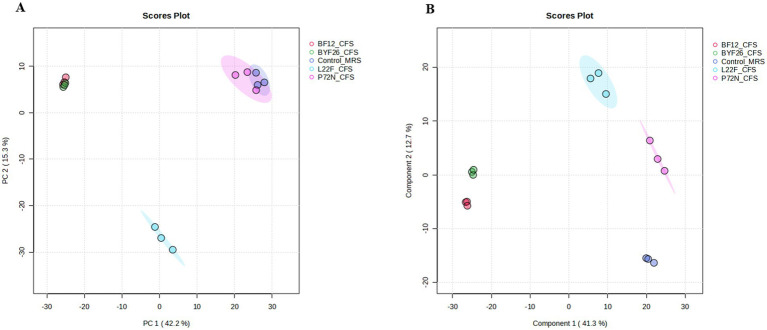
Principal component analysis, PCA **(A)** and partial least-squares discriminant analysis, PLS_DA **(B)** of the metabolite profile for the control_MRS and four sample groups. Cell free supernatant (CFS) of *L. salivarius* BF12 (BF12_CFS), CFS of *P. acidilactici* BYF26 (BYF26_CFS), CFS of *L. plantarum* L22F (L22F_CFS) and CFS of *P. acidilactici* P72N (P72N_CFS).

To identify the metabolites produced by each probiotic strain, metabolomic analyses were conducted on the CFSs. Using UHPLC-ESI-QTOF-MS, the principal component analysis (PCA) method was used to analyze among the control MRS group and sample groups (L22F_CFS, P72N_CFS, BF12_CFS and BYF26_CFS) to identify the overall situation of the metabolites. The PCA score plot displayed a clear separation between the control_MRS and sample groups across PC1 and PC2, which accounted for 42.2 and 15.3% of the total variance, respectively. Notably, the BF12_CFS vs. BYF26_CFS groups exhibit close clustering, whereas the L22F_CFS group was widely separated from the others. The control_MRS and P72N_CFS groups also exhibit close clustering (Figure 1A). To further identify the relevant metabolites responsible for group segregation, the partial least squares discriminant analysis (PLS-DA) based pairwise comparison method was used to show metabolomics differences. This identified distinct separation between the metabolic profiles of control_MRS and the sample groups which account for 41.3 and 12.7% of the total variance (Figure 1B). All PLS-DA models could be validated by the Response Permutation Test (RPT). Permutation test cross-validation was performed 1,000 times to ensure model suitability. The *p* value of the permutation test of the PLS-DA model was 0.009, and since it was <0.05 the null hypothesis was rejected, and the PLS-DA was valid in this study (Supplementary Figure S1).

#### Differential metabolites detection and class identification

**Figure 2:**
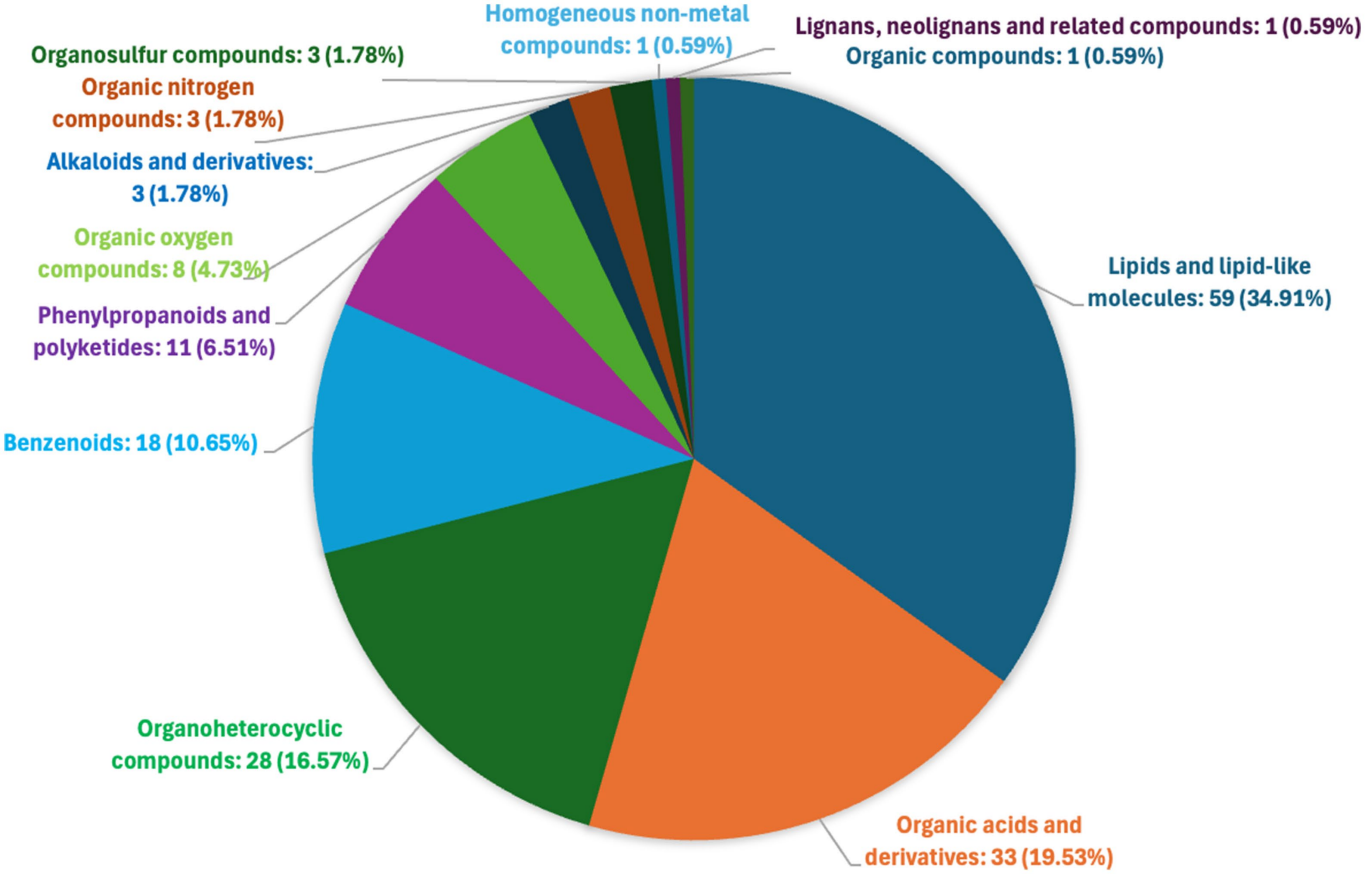
Classification of the metabolites (VIP > 2) based on human metabolome database (HMDB).

In total 1,330 metabolites in the sample group achieved statistical significance (*p* ≤ 0.01) with respect to the control media. Of these, a total of 169 metabolites (VIP > 2) were identified and confirmed in the Human Metabolome Database (HMDB) (Supplementary Table S4). The relative abundance of metabolites in the control-MRS and sample groups is shown in Figure 2, reflecting the content and intensity distribution of these metabolites. These compounds included: 59 lipids and lipid- like molecules (34.91%, fatty Acyls, glycerophospholipids, prenol lipids, steroids and steroid derivatives etc.); 33 organic acids and derivatives (19.53%, amino acids, peptides, and analogs; thiosulfinic acid esters; Hydroxy acids and derivatives etc.); 28 organoheterocyclic compounds (16.57%, azoles, indoles and derivatives; benzopyrans; pyridines and derivatives; heteroaromatic compounds etc.); 18 benzenoids (10.65%, anthracenes, fluorenes, naphthalenes, benzene and substituted derivatives etc.); 11 phenylpropanoids and polyketides (6.51%, 2-arylbenzofuran flavonoids, flavonoids, cinnamic acids and derivatives, macrolides and analogs etc.); 8 organic oxygen compounds (4.73%, organooxygen compounds); 3 alkaloids and derivatives (1.78%, harmala alkaloids, aspidospermatan-type alkaloids); 3 organic nitrogen compounds (1.78%, organonitrogen compounds); 3 organosulfur compounds (1.78%, organic trisulfides, sulfoxides, thiols); 1 homogeneous non-metal compounds (0.59%, halogen organides); 1 lignans, neolignans and related compounds (0.59%, dibenzylbutane lignans) and 1 organic compounds (0.59%, thiolactams) (Figure 2).

**Figure 3:**
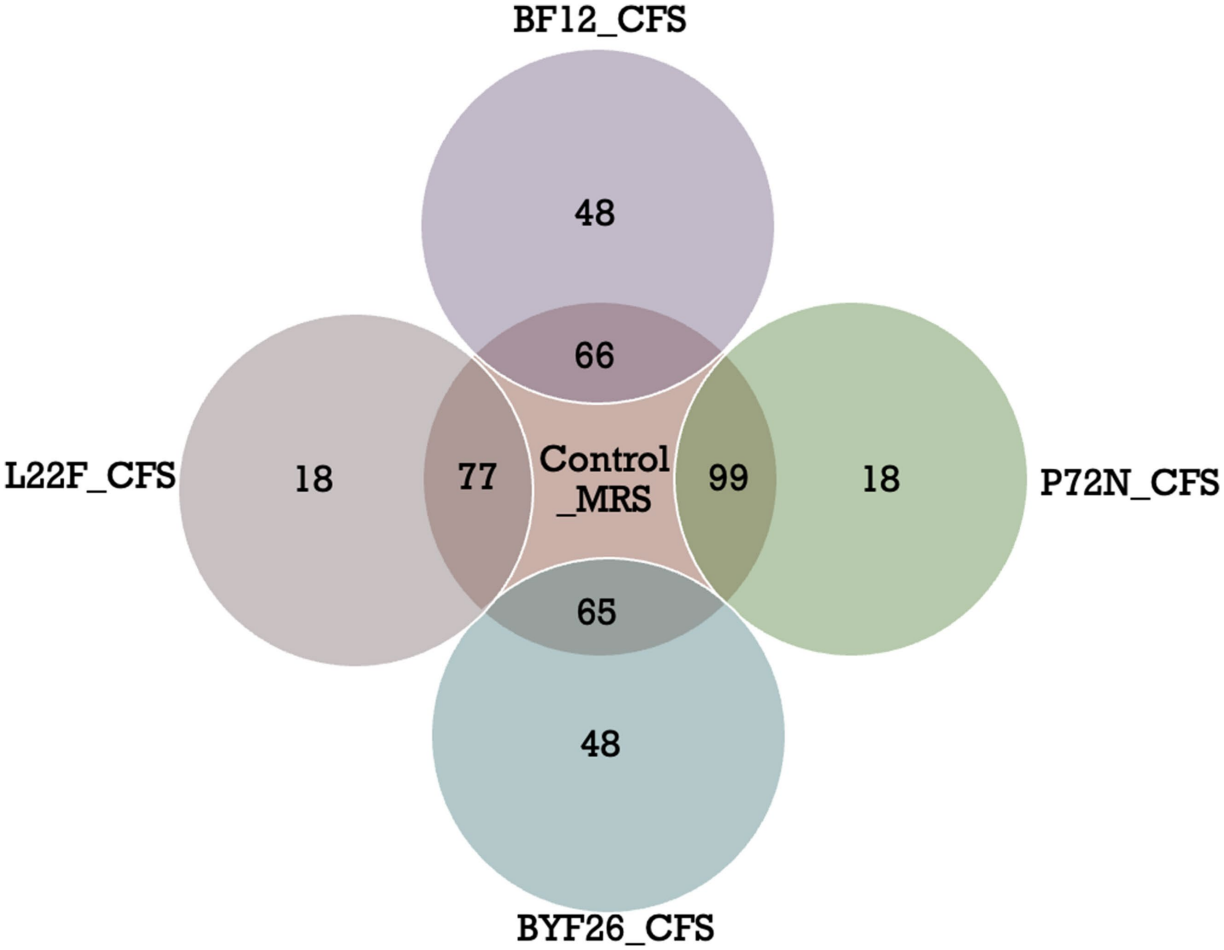
Venn diagrams of differential metabolites (VIP > 2) in control_MRS and sample groups. Cell free supernatant (CFS) of *L. salivarius* BF12 (BF12_CFS), CFS of *P. acidilactici* BYF26 (BYF26_CFS), CFS of *L. plantarum* L22F (L22F_CFS) and CFS of *P. acidilactici* P72N (P72N_CFS).

Among these compounds, there were a total of 120 metabolites in the control-MRS group, 95 in L22F-CFS, 117 in P72N-CFS, 114 in BF12 and 113 in BYF26. Some metabolites in the sample groups were shared with the control_MRS group, while the others were different. For better interpretation and visualization of the data, a Venn diagram showing shared and differential of metabolites between the control_MRS and sample groups is presented as Figure 3. There were 77 shared metabolites and 18 differential metabolites between the L22F_CFS and control_MRS groups, 99 shared and 18 differential metabolites between the P72N_CFS and control_MRS groups, 66 shared metabolites and 48 differential metabolites between the BF12_CFS and control_MRS groups, and 65 shared metabolites and 48 differential metabolites between the BYF26_CFS and control_MRS groups. The list of differential metabolites for each sample group are shown in Supplementary Table S5. The 18 differential metabolites in L22F_CFS included 8 organic acids and derivatives, 4 benzenoids, 2 phenylpropanoids and polyketides, 2 organoheterocyclic compounds, 1 lipids and lipid- like molecule and 1 organic oxygen compound. The 18 differential metabolites in P72N_CFS included 5 lipids and lipid- like molecules, 4 organic acids and derivatives, 4 benzenoids, 3 organoheterocyclic compounds, 1 organosulphur compound and 1 organic oxygen compound. BF12_CFS and BYF26_CFS included the same set of 48 differential metabolites comprising 14 lipids and lipid- like molecules, 11 organic acids and derivatives, 7 benzenoids, 7 organoheterocyclic compounds, 3 phenylpropanoids and polyketides, 2 organosulphur compounds, 1 alkaloids and derivatives, 1 lignans, neolignans and related compound, 1 organic compound and 1 organic oxygen compound (Supplementary Table S5).

#### Clustering heat map analysis of differential metabolites

**Figure 4:**
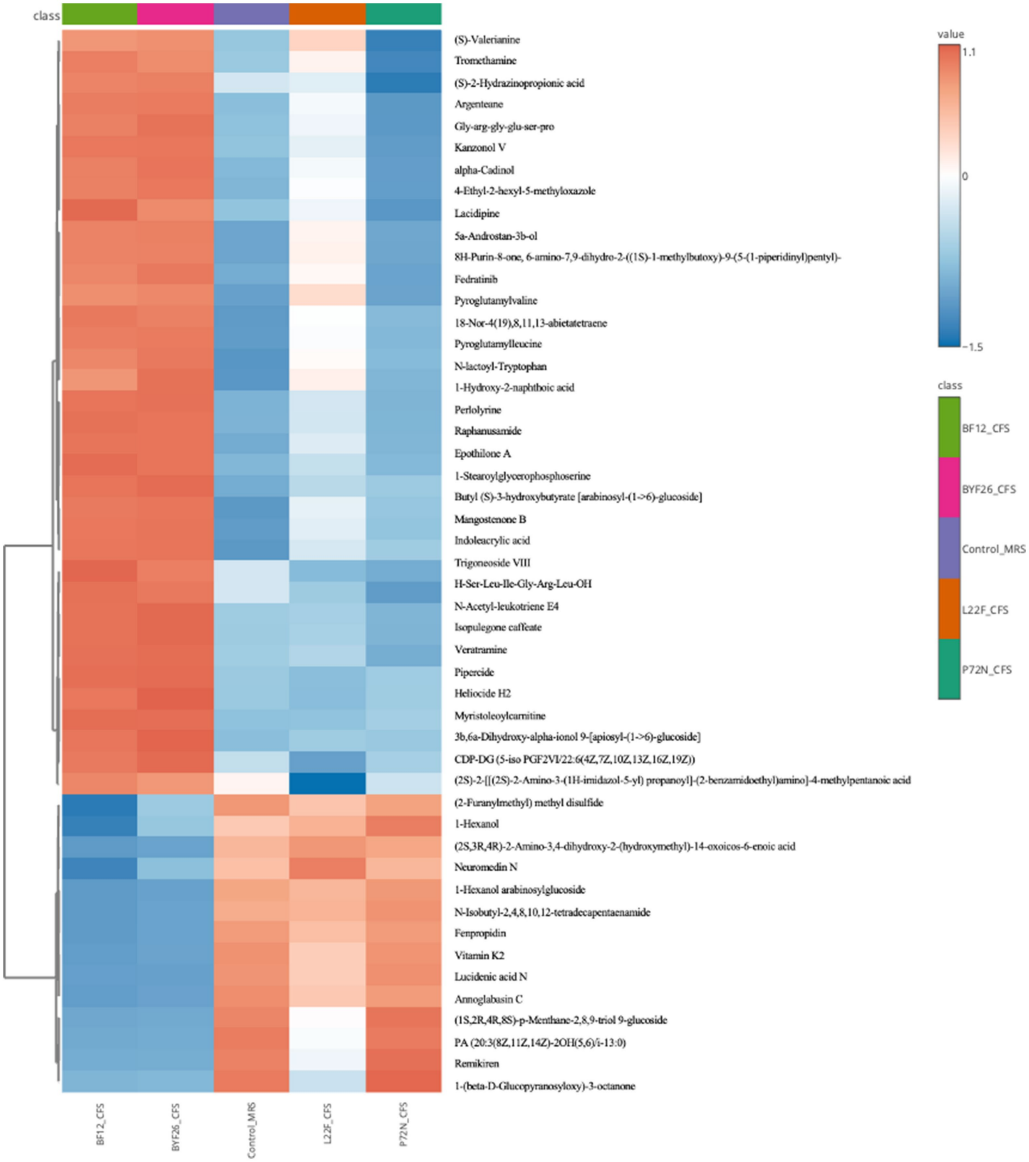
Heatmap for top 50 metabolites (VIP > 2).

A heatmap showing differences in the relative levels of the top 50 metabolites identified by the control_MRS and sample groups is shown as Figure 4. The L22F_CFS group exhibited a high content of lipids and lipid molecules, organic acids and derivatives, and phenylpropanoids and polyketides, with less common metabolites including 1 lipids and lipid molecule (Trigoneoside VIII), 4 organic acids and derivatives (Pyroglutamylleucine, Pyroglutamylvaline, N-lactoyl-Tryptophan, Gly-arg-gly-glu-ser-pro) and 2 phenylpropanoids and polyketides (Kanzonol V, Epothilone A) being up-regulated compared with the control_MRS group. BF12_CFS and BYF26_CFS had a similar pattern of metabolite differences. Both groups exhibited a high content of lipids and lipid molecules, organic acids and derivatives, organoheterocyclic compounds and phenylpropanoids and polyketides, with other less common compounds up-regulated compared to the control_MRS group including 6 lipids and lipid molecule (18-Nor-4(19),8,11,13-abietatetraene, 1-Stearoylglycerophosphoserine, Trigoneoside VIII, Butyl (S)-3-hydroxybutyrate [arabinosyl-(1- > 6)-glucoside], 3b,6a-Dihydroxy-alpha-ionol 9-[apiosyl-(1- > 6)-glucoside], 5a-Androstan-3b-ol), 4 organic acids and derivatives (Pyroglutamylleucine, Pyroglutamylvaline, N-lactoyl-Tryptophan, Gly-arg-gly-glu-ser-pro), 4 organoheterocyclic compounds (Indoleacrylic acid, Mangostenone B, 4-Ethyl-2-hexyl-5-methyloxazole, 8H-Purin-8-one, 6-amino-7,9-dihydro-2-((1S)-1-methylbutoxy)-9-(5-(1-piperidinyl)pentyl)-) and 2 phenylpropanoids and polyketides (Kanzonol V, Lacidipine). Compared with the control_MRS group the P72N_CFS group exhibited a high content of lipids and lipid molecules, organic acids and derivatives, and phenylpropanoids and polyketides, followed in the minority by 5 lipids and lipid molecule (1-Hexanol, 1-Hexanol arabinosylglucoside, 18-Nor-4(19),8,11,13-abietatetraene, Butyl (S)-3-hydroxybutyrate [arabinosyl-(1- > 6)-glucoside], Trigoneoside VIII), 1 organic acids and derivatives (N-lactoyl-Tryptophan) and 1 organoheterocyclic compounds (Indoleacrylic acid).

#### Specific bioactive metabolite detection inside the CFS of each probiotic strain

**Figure 5:**
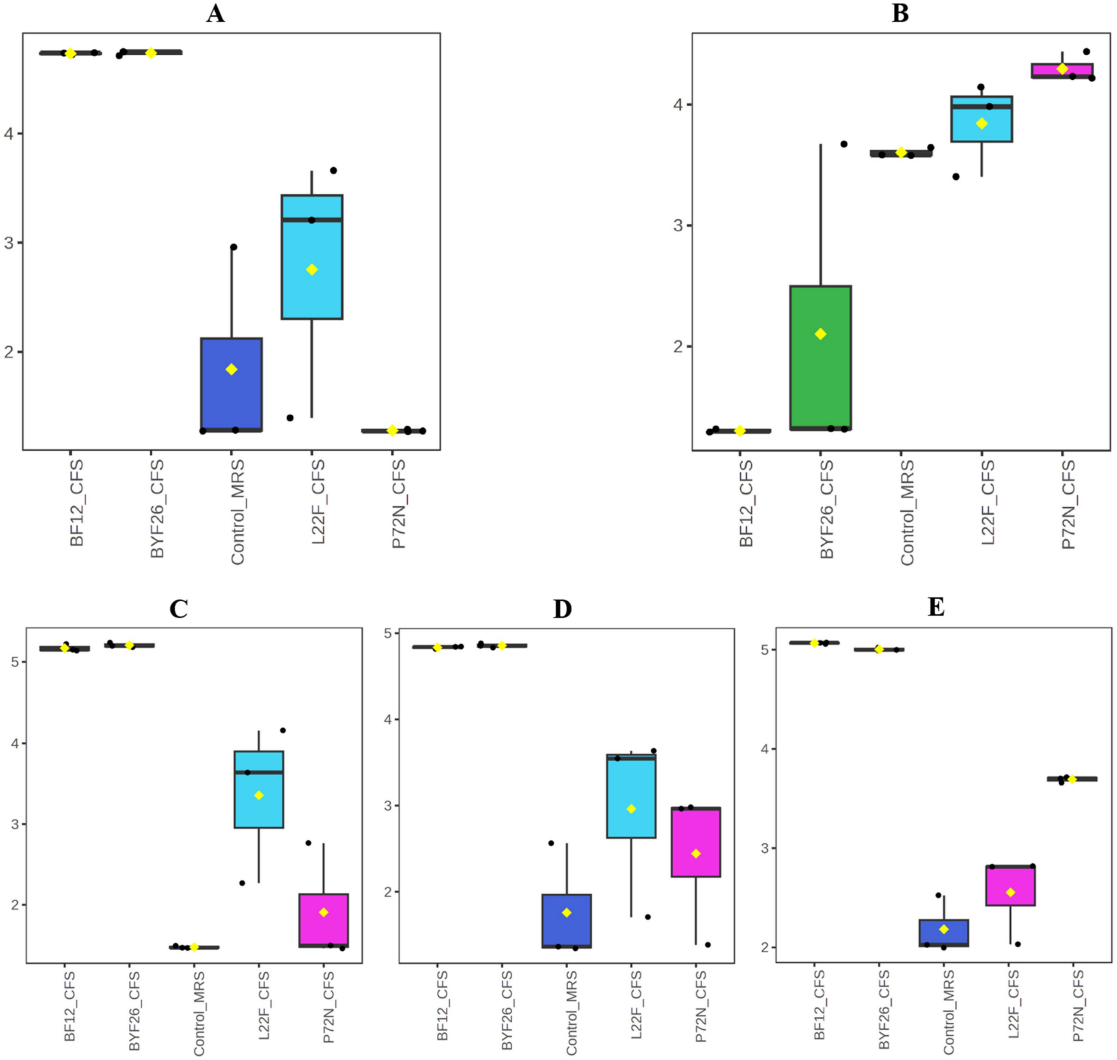
Boxplots of specific bioactive metabolites. Boxplots for Kanzonol V **(A)**, 1-Haxenol **(B)**, Pyroglutamylleucine **(C)**, Trigoneoside VIII **(D)** and 18-Nor-4(19),8,11,13-abietatetraene **(E)** are shown.

The range of the highlighted antibacterial metabolites that were unique to each strain is presented as a boxplot for individual compounds in Figure 5. The values displayed are from the metabolomic data that were normalized and scaled to the median value for each compound. Figure 5 shows that the levels of Kanzonol V were upregulated in L22F_CFS, BF12_CFS and BYF26_CFS groups (Figure 5A), whereas the levels of 1-Hexanol were significantly upregulated especially in the P72N_CFS group (Figure 5B). In addition, the levels of Pyroglutamylleucine were upregulated in the L22F_CFS, BF12_CFS and BYF26_CFS groups (Figure 5C). Trigoneoside VIII was abundant in all sample groups except the control_MRS group (Figure 5D), while 18-Nor-4 (19),8,11,13-abietatetraene was particularly enriched in P72N_CFS, BF12_CFS, and BYF26_CFS (Figure 5E).

### Physical characteristics of CFS-NLCs

The ingredients and composition of the CFS-NLCs from the selected probiotic candidates are shown in Table 2. The NLC formulation was carefully balanced, with a large proportion of bioactive CFS (79%) integrated within a lipid-based delivery system. The nano structure NLC production started by developing a nanostructured lipid blend. Medium triglycerides with Mantanov 82 were used as a solid lipid and were mixed with sorbitan oleate (span 80) as a liquid lipid, resulting in nanostructured lipid blends. The solid lipid formed the core structure, while the liquid lipid was integrated to disrupt crystallinity and enhance the CFS loading and release control. Tween 20 and poloxamer 188 then were added as the surfactant stabilizer reducing surface tension and preventing aggregation from the oil and aqueous phases to form NLCs. The biosurfactants in the cell free supernatant solubilize or emulsify lipids by embedding their hydrophobic tail into lipid molecules, or some might integrate into a lipid bilayer with their hydrophobic tail while the hydrophilic head remains exposed to the aqueous phase during amphiphilic interactions. The hydrophobic metabolites then may partition into the lipid core in NLCs to form a cell free supernatant encapsulated in a nanostructured lipid carrier (Table 2). The prototypes were creamy with a brownish color. CFS-NLCs separated and precipitated upon storage at 4°C. According to the DLS measurements, the mean nanoparticle size was 150.135 nm. The polydispersity index (PDI) was 0.245, and the zeta potential was −45.67 mV.

**Table 2:** The composition of nanostructured lipid carrier (NLCs) prototypes.

Phases	Components	Percentages	Notes
Oil phase	Medium-chain triglycerides	10	
	Montanov™ 82	2	Emulsifier
	Sorbitan Oleate (Span 80)	3	Emulsifier
Aqueous Phase	Polysorbate 20 (Tween 20)	3	Emulsifier
	Glycerin	2	Moisturizing agent
	Poloxamer 188	2	
	Phenoxyethanol	0.05	
	Cell-free supernatant (CFS)	79	
Sum		100% per mass	

### Antibacterial activity of the evaluated dose of CFS-NLCs

The antibacterial activity of the CFS-NLCs derived from the probiotic strains against the multi-drug-resistant pathogenic bacteria is shown in Table 3. The CFS-free NLCs showed no inhibition against any of the tested strains. However, all probiotic CFS-NLCs exhibited moderate inhibition against almost all *P. aeruginosa* strains. For the *S. pseudintermedius* strains, the CFS-NLCs exhibited moderate inhibition against the tested strains, except for P72N CFS-NLCs which showed moderate inhibition only against strains 159 and 130, while it displayed mild inhibition against strains 531, 668, and 998. Overall, the CFS-NLCs of all selected probiotic strains demonstrated moderate inhibition against the pathogenic bacteria tested.

**Table 3:** Antibacterial activity of CFS-NLCs of probiotic strains against pathogenic bacteria, *P. aeruginosa* and *S. pseudintermedius*.

LAB CFS	*P. aeruginosa*						*S. pseudintermedius*						*S. aureus*
	93	1846	1826	1,383	2054	ATCC27853	159	130	531	668	998	ED99	ATCC25923
CFS-free-NLCs	−	−	−	−	−	−	−	−	−	−	−	−	−
L22F CFS-NLCs	++	++	++	++	++	++	++	++	++	++	++	++	++
P72N CFS-NLCs	++	+	++	++	++	++	++	++	+	+	+	++	+
BF12 CFS-NLCs	++	++	+	++	++	++	++	++	++	++	++	++	++
BYF26 CFS-NLCs	++	++	++	++	++	++	++	++	++	++	++	++	+

The diameter of the inhibition zone was measured in millimeters and interpreted as (−) no inhibition, (+) mild inhibition (< 12 mm), (+ +) moderate inhibition (12–17 mm), (+ + +) strong inhibition (> 17 mm).

### Minimum bactericidal concentration of the evaluated dose CFS-NLCs

**Figure 6:**
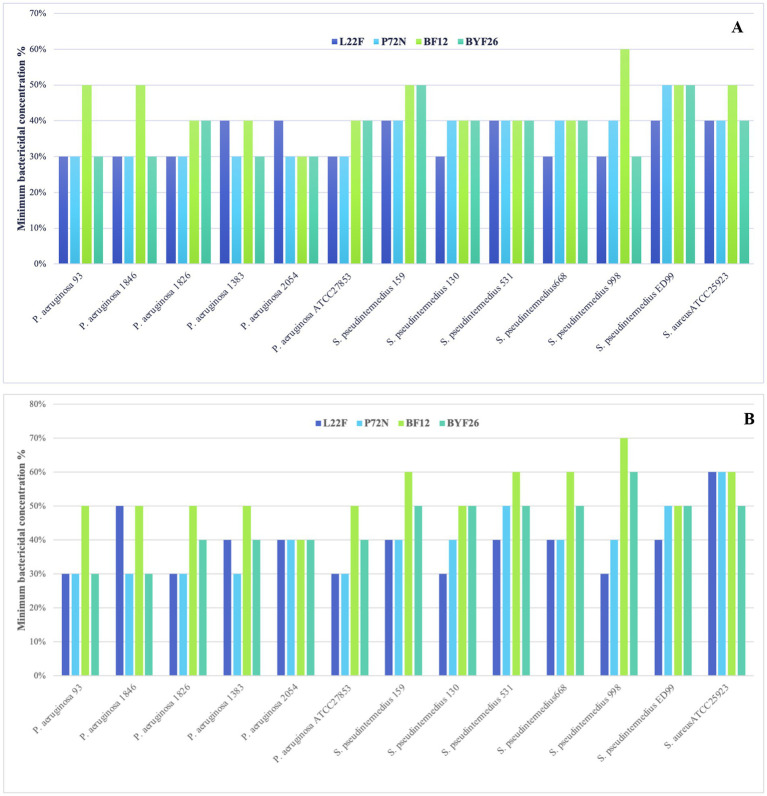
Minimum bactericidal concentration (MBC) calculated as a percent dilution of CFS-NLCs **(A)** and CFS-NLCs-3 month **(B)** produced from probiotic strains *L. plantarum* (L22F), *P. acidilactici* (P72N), *L. salivarius* (BF12) and *P. acidilactici* (BYF26) against multi-drug-resistant pathogens.

As anticipated, the CFS-free NLC emulsion did not exhibit antibacterial activity. The MBCs of the fresh CFS-NLCs and the CFS-NLCs after 3 months storage against multi-drug-resistant pathogens are shown in Figure 6. As shown in panel A, the MBCs of fresh CFS-NLCs derived from probiotic strains L22F, P72N and BYF26 displayed significant bactericidal activity across all *P. aeruginosa* and *S. pseudintermedius* strains, with percentages ranging between 30 and 50%. Notably, BF12 CFS-NLCs required the highest percentage (60%) to inhibit *S. pseudintermedius* 998. As shown in panel B, the bactericidal activity of CFS-NLCs after 3 months of storage remained within the range of 30–50%. However, slight changes were observed in L22F CFS-NLCs and BYF26 CFS-NLCs, which required a higher concentration (60%) to inhibit *S. pseudintermedius* 998 and *S. aureus* ATCC 25923. Notably, BF12 CFS-NLCs required percentages ranging from 40 to 60% to inhibit the pathogen strains, except for *S. pseudintermedius* 998 which needed the highest percentage (70%) for complete inhibition, suggesting a minor reduction in efficacy after prolonged storage. Overall, the CFS-NLCs maintained their antibacterial activity, with 30–60% dilutions effectively inhibiting the growth of pathogenic strains even after 3 months of storage.

## Discussion

This study investigated the antibacterial efficacy of probiotic-derived cell-free supernatants (CFS) encapsulated within nanostructured lipid carriers (NLCs) against multidrug-resistant pathogens commonly implicated in canine skin and ear infections, namely *P. aeruginosa* and *S. pseudintermedius*. The integration of metabolomic analysis offers novel insights into the bioactive compounds within these CFS formulations, and potential therapeutic application in veterinary medicine. Firstly, the antibacterial activities of the probiotic strains’ CFS against pathogenic bacteria were assessed using the agar well diffusion assay. The degree of inhibitory effect of the CFS from different probiotic strains to different indicator pathogen strains varied, particularly the CFS of *L. plantarum* (L25F) which did not show inhibitory effect on most *S. pseudintermedius* strains. This could be due to the strain-specific interaction, where the efficacy of the CFS is dependent on the type of pathogen targeted. Such strain-specific variations in inhibition have been noted in earlier studies, where probiotic effectiveness differed depending on the pathogen and the probiotic strain used. This underscores the importance of selection of probiotic strains for targeted antibacterial applications (19). We used acidified MRS (pH 3.8) to determine whether the inhibitory effect of our probiotic strains was solely due to the acidic conditions. Despite no inhibition being observed for the control MRS medium, acidified MRS showed mild inhibition against all pathogenic strains. Observation of the inhibition zone in the acidified MRS medium suggests that the acidic conditions alone can exert a slight inhibitory effect on pathogenic bacteria, consistent with findings from other studies that emphasize the role of pH in bacterial suppression (20). However, our CFS from the tested probiotic strains exhibit notable antibacterial activities against both *P. aeruginosa* and *S. pseudintermedius*, as evidenced by moderate inhibition zones. This implies that other metabolites produced by the probiotics, including bacteriocins or short-chain fatty acids, may be responsible for the heightened antibacterial activity. These findings are in agreement with previous reports indicating that probiotic strains, such as *Lactobacillus*, can produce a variety of antimicrobial substances that function synergistically with acidic conditions to inhibit pathogen growth (9, 20).

Probiotics can produce a wide range of bioactive compounds into the medium such as bacteriocins, organic acids, ethanol, diacetyl, carbon dioxide, and hydrogen peroxide (21). In the current study, we used liquid chromatography-mass spectrometry (LC–MS) to explore these bioactive compounds. Four probiotic candidates were selected for metabolomic analysis and further development of nano-encapsulation prototypes. PCA and PLS-DA are powerful statistical tools for analyzing inter-sample relationships in metabolomics. They are commonly used to assess and visualize overall metabolomic differences between samples (22). The PCA scores plot showed a clear separation between the control_MRS group and the sample groups, indicating distinct metabolic profiles for each group as evidenced by the separate clustering of the samples. Notably, the clustering of BF12_CFS and BYF26_CFS indicates similar metabolic profiles, while L22F_CFS is distinctly separated, highlighting its unique metabolic capabilities. In contrast, the close clustering of P72N_CFS with control_MRS suggests minimal metabolic differences between these two groups. The PLS-DA analysis revealed significant metabolic differences between the CFS groups and the control, implying that the strains produced a unique set of bioactive compounds. The PLS-DA model was further verified using a 1,000 random permutations test. The results showed that the PLS-DA model was valid and not overfitted since the *p* value was greater than 0.05, so the validated variable importance in projection (VIP) values were obtained from this model (23).

Through comprehensive metabolomic profiling, specific bioactive metabolites such as Kanzonol V and 1-Hexanol were identified as key components likely contributing to the CFS’s antibacterial efficacy. In addition, other beneficial compounds such as Pyroglutamylleucine, Trigoneoside VIII and 18-Nor-4 (19),8,11,13-abietatetraene which are likely to have anti-inflammatory, antimicrobial and antioxidant activities, were detected in the CFS. The identification of these metabolites marks a significant advance over previous studies that have broadly linked probiotic supernatants to antibacterial effects without characterizing the precise metabolic contributors. Kanzonols, well known prenylated flavonoids, are part of a group of phenolic compounds that exhibit antimicrobial properties (24). Sychrová et al., reported that prenylated flavonoids are potential therapeutic agents for the treatment of topical skin infections and wounds, as they can restore the balance in the wound microenvironment. Prenylated flavonoids are particularly effective in targeting bacterial cell membranes, leading to disruption of cell integrity and subsequent bacterial death (25). Although there is limited information about Kanzonol V’s detailed bioactivity, especially its antibacterial properties, antibacterial properties of Kanzonol C (26) and Kanzonol N (27) have been demonstrated, suggesting a potentially broad-spectrum antibacterial role for these flavonoids. As shown in Figure 5A, levels of Kanzonol V were upregulated in the sample groups except for the control and P72N_CFS groups. Therefore, Kanzonol V could be a potential antibacterial prenylated flavonoid compound for L22F_CFS, BF12_CFS and BYF26_CFS. On the other hand, Hexanol is a volatile alcohol and a major component of plant essential oils (EOs), with established antimicrobial effects. A study reported that the growth of *Escherichia coli* was delayed after over 3.9 mM of hexanol was directly added to liquid medium (28). Its mechanism of action involves interference with lipid synthesis and membrane integrity, leading to cell lysis. Interestingly, 1-Hexanol is selective in its activity, showing more potent effects against Gram-negative pathogens like *Pseudomonas aeruginosa*, which may explain part of the CFS’s efficacy against this pathogen (29). As shown in Figure 5B, the levels of 1-Hexanol were upregulated especially in P72N_CFS group. This could be the possible antibacterial compound in P72N_CFS acting against pathogenic bacteria. In addition, the levels of Pyroglutamylleucine were upregulated in the L22F_CFS, BF12_CFS, and BYF26_CFS groups (Figure 5C). Food protein hydrolysates and fermented foods contain pyroglutamyl peptides, which are spontaneously generated from peptides with a glutaminyl residue at the amino terminal during storage and processing. Pyroglutamylleucine is a form of peptide present in certain food protein hydrolysates. It is noteworthy for its function in modulating host immune responses, particularly by enhancing the production of endogenous antimicrobial peptides. While Pyroglutamylleucine does not have direct antibacterial effects, its role as an immune modulator may contribute indirectly to bacterial suppression by strengthening host defense mechanisms (30, 31). Moreover, this peptide showed an anti-inflammatory effect in chemically induced hepatitis in rats (32). Trigoneoside VIII is a furostanol saponin, which is structurally related to Trigoneoside VIII, which has demonstrated antimicrobial effects across various studies (33). 18-Nor-4 (19),8,11,13-abietatetraene is a diterpenoid, and it is structure suggests potential antioxidant capabilities, as diterpenoids can neutralize free radicals (34). The identification of bioactive compounds, particularly Kanzonol V, 1-Hexanol, Pyroglutamylleucine, Trigoneoside VIII and 18-Nor-4 (19),8,11,13-abietatetraene illustrates the effectiveness of using a metabolomics-guided approach to identify both antimicrobial, antioxidant and anti-inflammatory molecules in probiotic-derived CFS. These findings not only deepen our understanding of the specific mechanisms by which probiotics exert antimicrobial effects, but also highlight the potential of CFS as a source of natural bioactive compounds that can be harnessed for therapeutic applications. Encapsulating these metabolites within NLCs enhances their stability and sustained release, making them promising candidates for use in veterinary medicine to manage infections more sustainably and effectively.

NLCs are the product of nanotechnologies and are designed to transport hydrophilic and lipophilic chemicals or medications: they are primarily composed of solid lipid, liquid lipid, emulsifying agents, and counter-ions. This technology has recently been used in the cosmetic, pharmaceutical, and food industries for enhancing loading capacity, improving moisturizing agents, increasing bioavailability transmission via the skin, and prolonging release of chemicals (35). Moreover, nanoparticles are hydrophobic and therefore may be more efficiently absorbed by the lipid-rich epidermis and hair follicles (36–38). The prototype for NLCs, nano-emulsions, typically consists of several morphologies, including bicontinuous structures and swelling micelles that resemble droplets (39, 40). When lipids in nanostructures adhere to the skin, they create an adhesive film that obstructs the skin’s surface. Particles smaller than 400 nm have more pronounced occlusive effects (41). Simultaneously, the NLCs have a negative zeta potential that will prevent excessive skin penetration as they target bacterial skin pathogens (42). NLCs cannot be stored at room temperature long-term without sacrificing antibacterial efficacy (43); however, they separate and precipitate at 4°C. The nanoproduct droplet size as well as the uniformity of the droplet size and zeta potentials are related to product stability (44). Globule size is a critical parameter for assessing the performance of the emulsifying formulation; a smaller globule enables faster release rates and provides a larger interfacial surface area for drug absorption (45). The particle size of the NLC described here was below 200 nm, which is within the range of typical nano-emulsions (<400 nm) (46, 47); the PDI was 0.24 ± 0.02, which is below 0.3 and therefore homogeneous (48). A higher surface charge prevents droplet aggregation, which increases droplet size and decreases stability. The zeta potential of this product was more negative than -30 mV, and therefore it can be considered physically stable (47, 49). Ultimately, the properties of the NLCs described in this study satisfied several criteria that indicate efficacy and stability.

The antibacterial activity of CFS-NLCs derived from probiotic strains was rechecked against pathogenic bacteria to determine the maintenance of inhibitory activity. This result indicates that the antibacterial activity of the CFS was retained following nano-encapsulation, with enhanced efficacy observed, particularly against *S. pseudintermedius* strains, compared to the original CFS. This suggests that our nanostructured lipid carrier formulation did not negatively impact the antibacterial properties of the CFS and may even have enhanced its activity. Many studies have shown that nanostructured lipid carriers can protect sensitive bioactive compounds, allowing for sustained release and better penetration into bacterial biofilms or resistant bacterial strains (50, 51).

Antimicrobial activity is usually assessed by the determination of the MIC and MBC *in vitro*. The MBC is usually the same as the MIC for bactericidal drugs and generally is not more than four-fold higher (52). Considering only the MBC result, the bactericidal effect of CFS-NLCs from probiotic strains was demonstrated. Previous studies have shown that nanostructured lipid carriers can enhance the stability of encapsulated bioactive compounds, protecting them from degradation and preserving their activity over extended storage periods (53–55). In the present study, it was noticeable that the MBC percentage varied depending on formation and type of strains. Some formulations may require greater concentrations to achieve complete bacterial inhibition, which could be linked to differences in the bioactive compound profiles or different potency of antibacterial substances produced by different probiotic strains. Despite the promising results, a minor reduction in antibacterial efficacy in some formulations was observed after 3 months of storage, particularly in BF12 CFS-NLCs. This could be attributed to gradual degradation of the bioactive compounds or a slight loss of efficacy due to storage conditions. Variables such as temperature, humidity, and exposure to light can accelerate the degradation processes of both the lipid carriers and the encapsulated compounds. A study on *β*-carotene-loaded NLCs observed that storage at higher temperatures led to significant degradation of β-carotene, impacting the formulation’s efficacy (56). Overall, the ability of CFS-NLCs to retain bactericidal efficacy over time, even at dilutions between 30 and 60%, underscores the robustness of the nano-encapsulation approach.

In conclusion, this study underscores the potential of nanostructured lipid carriers (NLCs) encapsulating probiotic-derived cell-free supernatants (CFS) as effective antibacterial agents against multidrug-resistant pathogens associated with canine dermatitis and otitis. Integrating metabolomic profiling added a novel dimension to our findings, identifying specific bioactive compounds, such as Kanzonol V and 1-Hexanol, contributing to the antibacterial efficacy. Unlike prior studies that broadly attribute antimicrobial properties to probiotic supernatants, our metabolomic analysis allowed for precisely identifying these compounds, highlighting unique metabolic signatures that enhance pathogen inhibition. Even after prolonged storage, the sustained antibacterial activity observed in CFS-NLCs demonstrates the formulation’s potential for use in veterinary therapeutics. Despite these promising results, several limitations warrant attention. The *in vitro* nature of this study does not account for the complexities of *in vivo* environments, where metabolite stability and bioavailability may vary. Future research should focus on optimizing storage conditions and conducting *in vivo* studies to further validate the therapeutic potential of CFS-NLCs for the treatment of infections, particularly in veterinary applications.

## Data Availability

The original contributions presented in the study are included in the article/Supplementary material, further inquiries can be directed to the corresponding author.

## Footnotes

^1^ https://www.metaboanalyst.ca
