# Supplementary material for: Metabolomic profiling and antibacterial efficacy of probiotic-derived cell-free supernatant encapsulated in nanostructured lipid carriers against canine multidrug-resistant bacteria
Source: Front Vet Sci. 2025 Jan 3;11:1525897. doi: 10.3389/fvets.2024.1525897 (PMC11739306; doi:10.3389/fvets.2024.1525897)
Supplement: Supplementary file 1 [file Data_Sheet_1.docx]

**Supplementary data**

**Supplementary Table 1.** Sources of samples and host species of origin of the two pathogenic bacteria

| Pathogenic bacteria | Strains | Sources of samples | Species of Origin |
| --- | --- | --- | --- |
| *Pseudomonas aeruginosa* | 93 | Open wound | Dog |
|  | 1846 | Open Wound | Cat |
|  | 1826 | Exudate from Wound | Dog |
|  | 1383 | Abscess | Dog |
|  | 2054 | Open wound | Dog |
| *Staphylococcus aureus* | 159 | Pustule | Dog |
|  | 130 | Exudate From Wound | Dog |
|  | 531 | Exudate From Wound | Dog |
|  | 668 | Wound | Dog |
|  | 998 | Chronic Wound | Dog |

**Supplementary Table 2.** Results for antimicrobial susceptibility testing (antibiogram) of five *S. pseudintermedius* strains determined in the VITEK 2® system.

| Antimicrobials | MIC (µg/ml) | | | | |
| --- | --- | --- | --- | --- | --- |
|  | *S. pseudintermedius* strains | | | | |
|  | 130 | 159 | 531 | 668 | 998 |
| Cefoxitin screen | POS (+) | NEG (-) | POS (+) | POS (+) | POS (+) |
| Benzylpenicillin | ≥ 0.5 (R) | ≥ 0.5 (R) | ≥ 0.5 (R) | ≥ 0.5 (R) | ≥ 0.5 (R) |
| Amoxicillin/Clavulanic acid | 8 (R) | ≤ 2 (S) | 8 (R) | 8 (R) | 8 (R) |
| Oxacillin | ≥ 4 (R) | ≤ 0.25 (S) | ≥ 4 (R) | ≥ 4 (R) | ≥ 4 (R) |
| Cefalotin | ≥ 32 (R) | ≤ 2 (S) | ≥ 32 (R) | ≥ 32 (R) | ≤ 2 (R) |
| Cefpodoxime | ≥ 8 (R) | ≤ 0.5 (S) | ≥ 8 (R) | ≥ 8 (R) | ≥ 8 (R) |
| Cefovecin | ≥ 8 (R) | ≤ 0.5 (S) | ≥ 8 (R) | ≥ 8 (R) | ≥ 8 (R) |
| Gentamicin | ≥ 16 (R) | 8 (I) | ≥ 16 (R) | ≥ 16 (R) | 8 (I) |
| Enrofloxacin | ≥ 4 (R) | 1 (I) | ≥ 4 (R) | ≥ 4 (R) | ≥ 4 (R) |
| Marbofloxacin | ≥ 4 (R) | 1 (S) | ≥ 4 (R) | ≥ 4 (R) | ≥ 4 (R) |
| Inducible Clindamycin Resistance | NEG (-) | NEG (-) | NEG (-) | NEG (-) | NEG (-) |
| Erythromycin | ≥ 8 (R) | 0.5 (S) | ≥ 8 (R) | ≥ 8 (R) | ≥ 8 (R) |
| Clindamycin | ≥ 4 (R) | 0.25 (S) | ≥ 4 (R) | ≥ 4 (R) | ≥ 4 (R) |
| Vancomycin | ≤ 0.5 (S) | ≤ 0.5 (S) | 1 (S) | 1 (S) | ≤ 0.5 (S) |
| Tetracycline | ≥ 16 (R) | ≤ 1 (R) | ≥ 16 (R) | ≥ 16 (R) | ≥ 16 (R) |
| Nitrofurantoin | ≤ 16 (S) | ≤ 16 (S) | ≤ 16 (S) | ≤ 16 (S) | ≤ 16 (S) |
| Fusidic acid | ≤ 0.5 (S) | ≤ 0.5 (S) | ≤ 0.5 (S) | ≤ 0.5 (S) | ≤ 0.5 (S) |
| Mupirocin | ≥ 512 (R) |  |  | ≤ 1 (S) | 32 (I) |
| Chloramphenicol | 8 (S) | 8 (S) | 8 (S) | ≥ 64 (R) | 8 (S) |
| Rifampicin | ≤ 0.5 (S) | ≤ 0.5 (S) | ≤ 0.5 (S) | ≤ 0.5 (S) | ≤ 0.5 (S) |
| Trimethoprim/Sulfamethoxazole | ≥ 320 (R) | ≥ 320 (R) | ≥ 320 (R) | ≤ 10 (R) | ≥ 320 (R) |

Abbreviations; S, susceptible; I, intermediate; R, resistant; +, positive; -, negative

**Supplementary Table 3.** Results for antimicrobial susceptibility testing (antibiogram) of seven *P. aeruginosa* strains determined in the VITEK 2 system.

| Antimicrobials | MIC (µg/ml) | | | | | | |
| --- | --- | --- | --- | --- | --- | --- | --- |
|  | *Ps. aeruginosa* strains | | | | | | |
|  | 93 | M1309 | M1241 | 1383 | 1826 | 1846 | 2054 |
| Piperacillin |  |  |  | ≥ 128 (R) | 8 (S) | 8 (S) | 16 (S) |
| Imipenem | ≥ 16 (R) | ≥ 16 (R) | 2 (S) | 8 (R) | 4 (I) | 8 (R) | 4 (I) |
| Amikacin | 4 (S) | ≤ 2 (S) | ≥ 64 (R) | ≤ 2 (S) | ≤ 2 (S) | ≤ 2 (S) | ≤ 2 (S) |
| Gentamicin | ≤ 1 (S) | ≤ 1 (S) | ≥ 16 (R) | ≤ 1 (S) | ≤ 1 (S) | ≤ 1 (S) | ≤ 1 (S) |
| Tobramycin |  |  |  | ≤ 1 (S) | ≤ 1 (S) | ≤ 1 (S) | ≤ 1 (S) |
| Enrofloxacin | 1 (I) | 2 (I) | ≥ 4 (R) | 2 (I) | 2 (I) | 1 (I) | 1 (I) |
| Marbofloxacin | ≤ 0.5 (S) | ≤ 0.5 (S) | ≥ 4 (R) | ≤ 0.5 (S) | ≤ 0.5 (S) | ≤ 0.5 (S) | ≤ 0.5 (S) |
| Polymyxin B |  |  |  | 2 | 1 | 2 | 1 |

Abbreviations; S, susceptible; I, intermediate; R, resistant.

**Supplementary Table 4.** Significant metabolites (VIP>2) confirmed in the Human Metabolome Database (HMDB).

**Supplementary Table 5.** Differential metabolites in each sample group (L22F_CFS, P72N_CFS, BF12_CFS and BYF26_CFS).


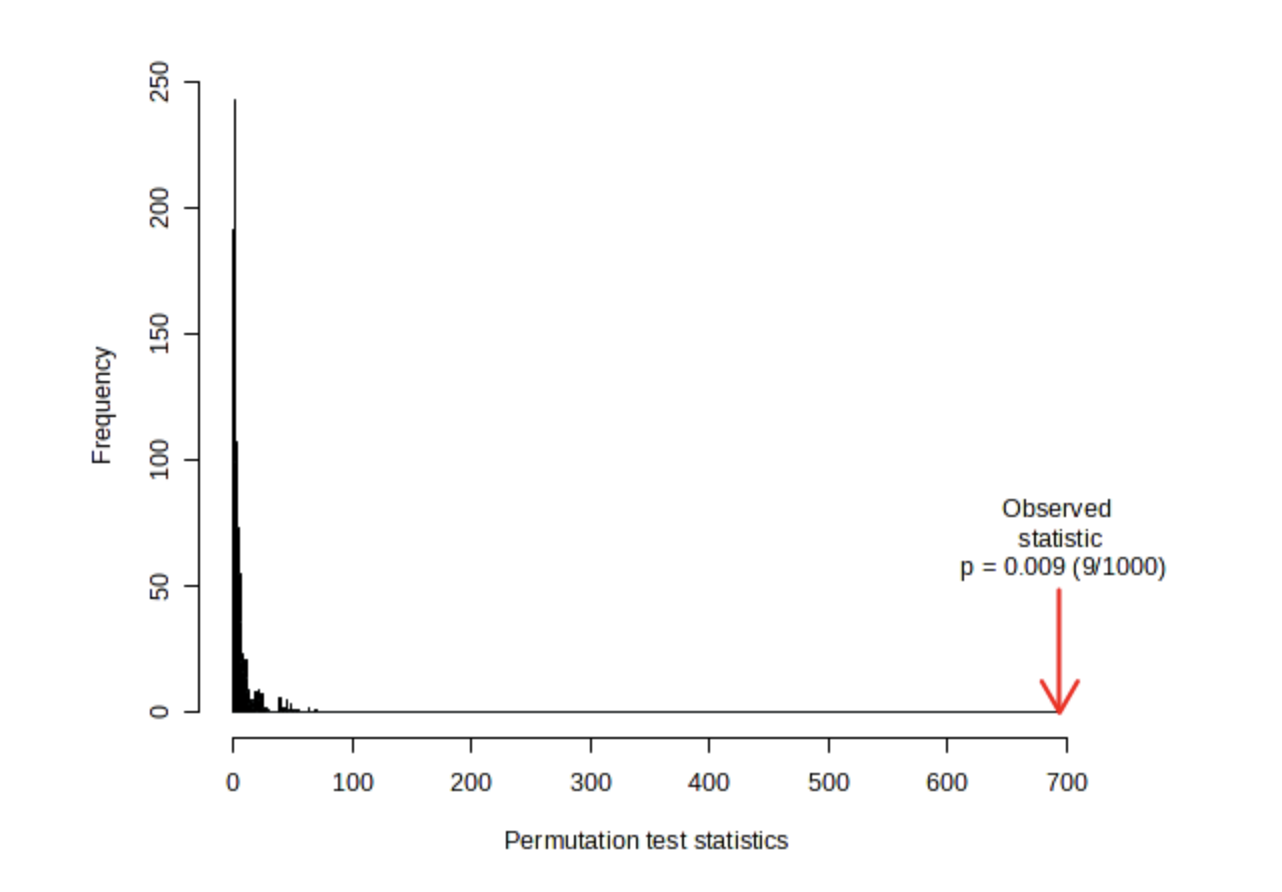


**Supplementary Figure 1.** Permutation test statistics results with 1000 random permutations based on the PLS-DA model
